# Not everyone is special: evaluation of indication in a urinalysis-driven reflex urine culture protocol at an academic medical center

**DOI:** 10.1017/ice.2026.10419

**Published:** 2026-04

**Authors:** Mackenzie Rae Keintz, Jasmine R. Marcelin, Mark E. Rupp, Trevor C. Van Schooneveld

**Affiliations:** 1 Division of Infectious Disease, University of Nebraska Medical Center, Omaha, NE, USA

## Abstract

Asymptomatic bacteriuria (ASB) frequently results in inappropriate antimicrobial use. Urinalysis (UA)-driven reflex urine culture order sets can reduce inappropriate urine cultures. Most special indications on free text overrides within a (UA)-driven reflex culture protocol were inappropriately defined and rarely symptom-based. Eliminating the “other” indication could strengthen diagnostic stewardship and reduce unnecessary urine cultures.

## Introduction

**Figure 1.**
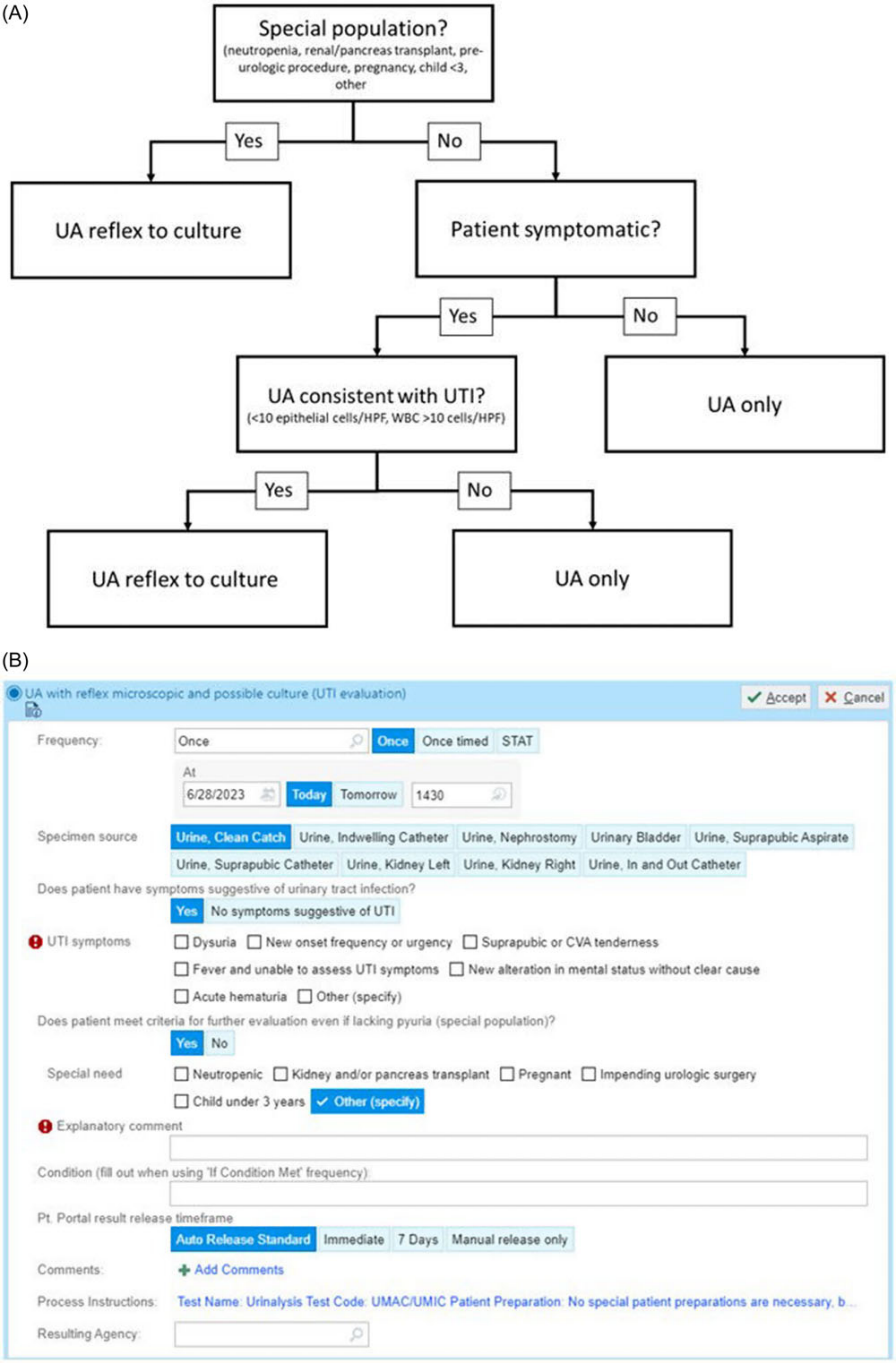
(A) Description of laboratory decision pathway for symptom/ (UA) driven urine culture algorithm. (B) UA drive reflex urine culture order set screenshot. UTI, urinary tract infection; HPF, high powered field.

Asymptomatic bacteriuria (ASB) is common in hospitalized patients and detection often results in inappropriate antimicrobial treatment which can result in harm.^1^ Reducing urine cultures can help decrease inappropriate treatment of ASB as well as decreasing inappropriate diagnosis of catheter associated urinary tract infection.^2,3^ Implementation of a reflex urine culture protocol has had mixed results in the literature in reducing the number of urine cultures and subsequent antimicrobial treatment due to poor correlation of urinalysis (UA) results with the diagnosis of urinary tract infections (UTI).^4–6^ In 2014, our institution implemented a mandatory UA-driven reflex culture orderset predicated on symptoms, UA results, and patient risk factors (Figure 1A). We previously demonstrated a reduction in urine cultures with this approach.^3^ In our orderset, clinicians have a field to document urinary symptoms based on institutional guidance: dysuria, new onset frequency or urgency, suprapubic or CVA tenderness, fever and inability to assess UTI symptoms, acute hematuria, new alteration in mental status without clear cause, and other (specify). It also included a “special” designation field allowing clinicians to override the reflex algorithm when patients had conditions in which treatment of ASB may be indicated or when symptoms and UA findings may be unreliable (Figure 1B). Selecting “special” bypassed the UA-based criteria and prompted a urine culture. These risk factors and conditions included pregnancy, renal/pancreas transplant, age <3, neutropenia, impending urologic procedure, and an “other” free text indication. If symptoms were present and no “special” indication was designated, the algorithm assessed for UA results including pyuria (>10 WBC/HPF) and epithelial cells (<10 epithelial cells/ HPF). If all criteria were met, a urine culture was performed.

We evaluated the special population “other” free text option to determine if additional indications were needed and if data entered was medically appropriate.

## Methods

This retrospective review at a tertiary academic medical center included all inpatient UA-driven reflex urine cultures ordered between 7/1/2020–6/30/2022. UA and urine cultures obtained in the emergency department or ambulatory care setting were excluded due to the reflex orderset not being implemented in these settings. Descriptive statistics were used to evaluate orderset utilization, with a particular focus on the “other” free-text indication, which was analyzed to assess medical appropriateness.

## Results

**Figure 2.**
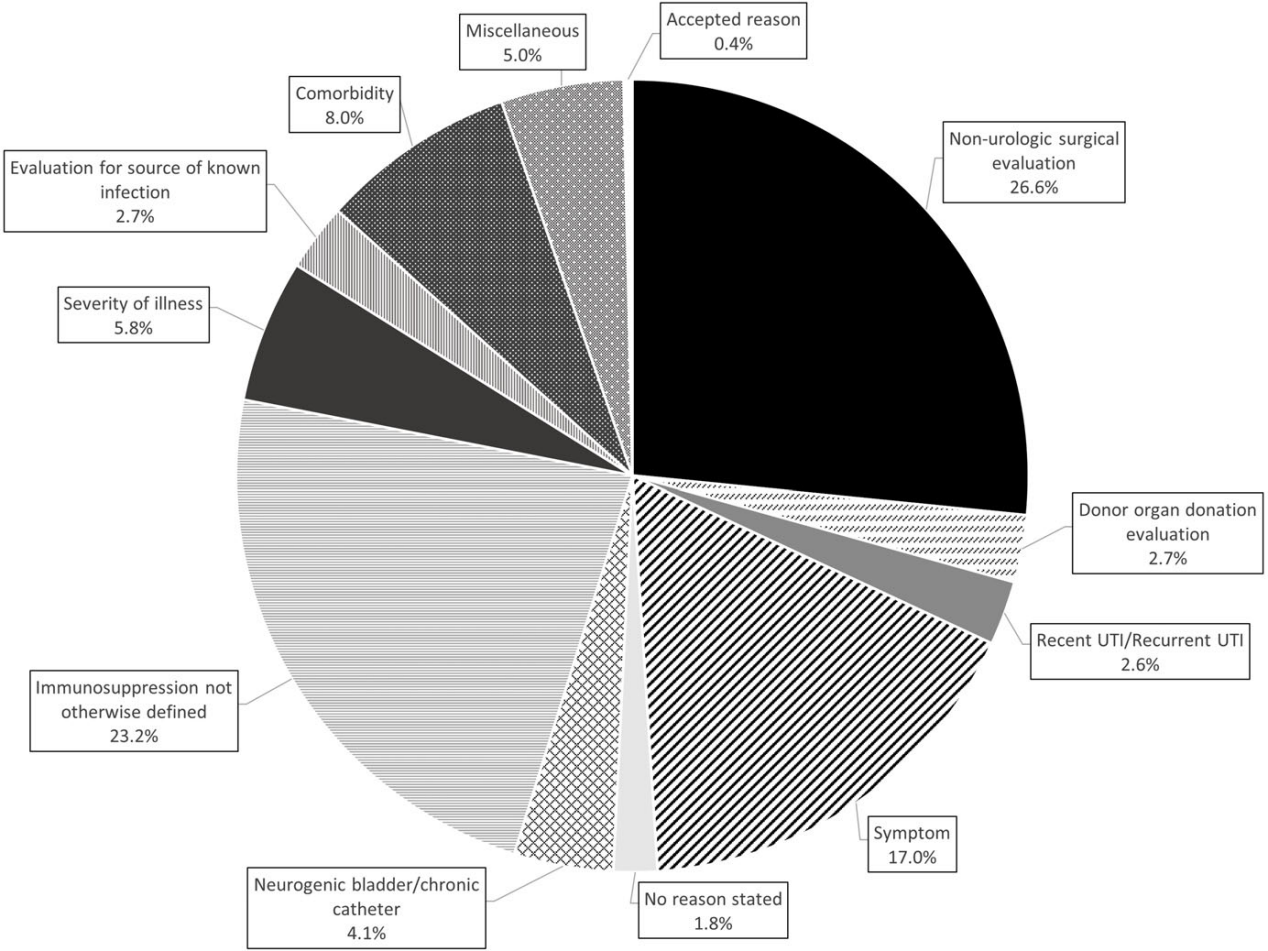
Distribution of “other” free text indication by theme (*N* = 839).

A total of 35,469 UA-driven reflex culture order sets were submitted, of which 26.8% (*N* = 9,493) resulted in culture. Among the UA-driven reflex culture order sets, 2,085 (5.8%) included a selected “special” indication, which bypassed the reflex algorithm and triggered an automatic culture. “Other” was the most cited indication for special population override, contributing to 40% (*n* = 839/2085) of these indications, followed by renal/pancreas transplant (29%) and neutropenia (13%). The “other” free text options fell into one of 11 themes (Figure 2). The three most common reasons a urine culture was obtained using the “other” free text option were non-urologic surgical intervention 26.6% (*n* = 223/839), immunosuppression not otherwise defined 23.2% (*n* = 195/839), and redocumenting urinary or systemic symptoms 17.4% (*n* = 146/839). Based on current literature, nearly all “other” indications were inappropriate for bypassing the reflex protocol (*n* = 816/839). Only 52% (*n* = 428/839) of patients in which the “other” indication was selected had urinary symptoms documented in the order set. If the UTI protocol had been strictly followed, 696/839 (83%) cultures ordered with an indication of “other” would not have been obtained, due to either lack of symptoms or if symptomatic, lack of pyuria. Of these, 73 cultures identified>100,000 colony forming units and 296 cultures identified bacteria not meeting the definition of bacteriuria.

## Discussion

Diagnostic stewardship is an essential tool to guide appropriate test use and avoid patient harm through treatment of ASB. Electronic diagnostic stewardship efforts should take into account possible medically appropriate reasons to bypass guidance. In addition, these strategies should be re-evaluated periodically to determine if efficiencies can be added or if misuse is occurring.^7^ We found most cultures obtained by selecting the “other” free text indication to override the urine culture protocol were inappropriate, highlighting potential improvements to UA-driven reflex culture order set. Although the free-text option was intended to accommodate uncommon but clinically justified scenarios, our analysis shows it was frequently misused. Appropriately justified use of the free-text option generally reflected documentation of an accepted risk factor already available within the structured order set (e.g., neutropenia, pregnancy, age <3 years, renal/pancreas transplant, or upcoming urologic procedure) or deceased donor evaluation for potential organ transplantation per regulatory requirements. Other indications including immunosuppression not otherwise specified, non-urologic procedures, and severity of illness do not directly necessitate that urine culture is obtained. In particular, non-urologic procedures which were primarily cardiac and orthopedic surgeries are not supported by current literature.^8^

Only about half of patients with an “other” indication had documented urinary symptoms, and a substantial proportion lacked pyuria even when symptoms were present. This pattern highlights a tendency to obtain cultures based on factors other than clinical presentation of UTI, increasing the likelihood of detecting and subsequently treating ASB. The high proportion of such cultures underscores the vulnerability of reflex testing protocols to overuse when override pathways are overly permissive.

Analysis of the free-text indications suggests that eliminating the “other” option would not meaningfully increase the risk of missing appropriate cultures. All clinically valid indications identified within the free-text entries are either already available as structured selections within the order set or represent a single, clearly defined need, i.e., deceased donor evaluation, that can be easily incorporated as its own indication to support regulatory requirements. Clinicians may appeal algorithm-based decisions by contacting the antimicrobial stewardship medical director when clinical judgement indicates a urine culture may be appropriate. Restricting the free-text option therefore represents a low-risk, high-yield modification to strengthen adherence to evidence-based testing criteria. A potential consequence of removing the free-text option is substitution with an inappropriate structured indication to obtain culture. To address this concern, postimplementation monitoring of “special” indication selection is ongoing. These findings reveal an opportunity to improve diagnostic stewardship through targeted revisions to the order-set structure.
